# Wilson’s Disease Myopathy: A Case Report and Brief Literature Review

**DOI:** 10.7759/cureus.99405

**Published:** 2025-12-16

**Authors:** Nicholas M Riccione, Cassie N Chan

**Affiliations:** 1 Neurology, University of Texas Health Science Center at San Antonio, San Antonio, USA

**Keywords:** myopathy, neurology, neurology outpatient care, neuromuscular diseases, wilson's disease

## Abstract

Wilson’s disease is a rare genetic disorder of copper toxicity with known systemic, multiorgan effects. The spectrum of symptoms is wide, ranging from relatively common and well-described central neurologic involvement to sparse case reports describing peripheral neuromuscular involvement, particularly as a primary presenting symptom. We describe a case involving a convoluted diagnostic course that ultimately led to a diagnosis of myopathy attributed to newly diagnosed Wilson’s disease in a 37-year-old male. We also provide a literature review of similar cases and proposed pathophysiologic mechanisms, consolidating current knowledge on the spectrum of musculoskeletal symptoms associated with Wilson’s disease. This case illustrates the importance of considering Wilson’s disease in cases of peripheral idiopathic myopathies, especially when comorbidities may be consolidated to suggest a single overarching causative diagnosis.

## Introduction

Wilson’s disease is an autosomal recessive disorder of copper metabolism first defined in 1912 that causes dysfunction of the intracellular copper transporter ATP7B and subsequent excessive copper accumulation in multiple organ systems, primarily the liver, eyes, and brain [1]. It has traditionally been viewed within the neurologic sphere as a secondary movement disorder, in which copper deposition in various brain regions, most commonly the basal ganglia, thalamus, cerebellum, and upper brainstem, causes a wide range of neurologic symptoms of central origin [2]. Copper has a vital function in the body as an enzyme cofactor; however, in Wilson’s disease, dysfunctional ATP7B results in an impaired ability to excrete excess copper into bile in the liver, with subsequent release of free copper into the bloodstream unbound to ceruloplasmin and free to deposit throughout the body [3]. The resulting elevated copper levels can catalyze the formation of reactive hydroxyl radicals, thereby causing oxidative damage [4]. This article presents a rare case of Wilson’s disease-associated myopathy and details similar rare cases in an effort to consolidate the limited existing knowledge of this pathology.

## Case presentation

A 36-year-old male with a prior history of presumed alcoholic cirrhosis and chronic lower back pain presented for evaluation of progressive weakness and ptosis in February 2025. Approximately one year earlier, he first noted reduced bilateral grip strength, with subsequent progression to a slowed gait, requiring ambulation with a cane and walker due to cramping when walking for prolonged periods. His progressive weakness was painless and associated with an unintentional 30-pound weight loss, as well as new-onset significant anxiety that reduced his nighttime sleep. The patient’s wife noted progressive slurred speech, with observed choking on water and even his own saliva. The patient reported jaw cramping with eating but no fatigue with chewing. He denied associated blurry vision, ptosis, or diplopia.

He subsequently presented to a local emergency department for evaluation and underwent MRI of the brain, cervical spine, and thoracic spine, as well as CT angiography of the head and neck, all of which were unremarkable. He was discharged and later underwent outpatient evaluation, which revealed moderate degenerative changes on MRI of the lumbar spine and a negative myasthenia gravis antibody panel. A brief trial of pyridostigmine (60 mg) was performed without benefit. Six sessions of plasma exchange and a course of IVIG (2 g/kg) were also trialed without symptom improvement. Interval CT of the chest showed no thymic abnormalities. Given a largely negative workup despite high clinical suspicion, the patient was referred to a neuromuscular specialist, with a planned interim single-fiber EMG for presumed seronegative myasthenia gravis. Additional serum studies performed over several months by multiple providers, including antinuclear antibody, HbA1c, thyroid-stimulating hormone, HIV, Lyme, Treponema antibodies, vitamin B12, folate, thiamine, heavy metals, a ganglioside panel, and serum protein electrophoresis with immunofixation, were within normal limits (Table 1, Table 2), except for a slightly elevated angiotensin-converting enzyme level (as reported by an outside hospital, without an exact value available).

**Table 1:** Composite of serum and urine quantitative testing performed This table lists all available serum and urine quantitative values obtained during the patient workup. All studies listed are serum unless otherwise specified in the study name. TSH, thyroid-stimulating hormone

Serum study	Value	Reference range
TSH	1.457 µIU/mL	0.350-4.940 µIU/mL
Vitamin B12	>2000 pg/mL	213-816 pg/mL
Vitamin E	8.6 mg/L	5.5-18.0 mg/L
Folate	10.2 ng/mL	7.0-31.4 ng/mL
Arsenic	<10.0 µg/L	<12.0 µg/L
Lead	<2.0 µg/dL	<4.9 µg/dL
Mercury	<2.5 µg/L	<10.0 µg/L
GM1 antibody	23 IV	0-50 IV
GM2 antibody	11 IV	0-50 IV
GD1a antibody	11 IV	0-50 IV
GD1bL antibody	11 IV	0-50 IV
GQ1b antibody	8 IV	0-50 IV
Acetylcholine receptor ganglionic (α3) antibody	<55 pmol/L	<55 pmol/L
Lactate dehydrogenase	111 U/L	125-275 U/L
Creatine kinase	32 U/L	30-200 U/L
Aldolase	3.5 U/L	<8.1 U/L
Acetylcholine-binding antibody	0 nmol/L	0-0.4 nmol/L
Acetylcholine-blocking antibody	0%	0-26%
Voltage-gated calcium channel antibody	<30 pmol/L	<30 pmol/L
24-hour urine copper	956 µg/24 h	15-60 µg/24 h
Ceruloplasmin	7 mg/dL	14-30 mg/dL

**Table 2:** Composite of serum qualitative laboratory testing performed This table lists all available serum qualitative values obtained during the patient workup. ANA, antinuclear antibody

Serum study	Result
ANA	Negative
HIV	Nonreactive
Lyme	Negative
Treponema	Negative
Anti-Hu antibody	Negative
ANNA-2 antibody	Negative
PCA-1 (Yo) antibody	Negative
PCA-2 antibody	Negative
AGNA/SOX1 antibody	Negative
Amphiphysin antibody	Negative
CRMP5/CV2 antibody	Negative
Striated muscle antibody	Negative
211-gene genetic neuromuscular panel	Homozygous pathogenic variant of ATP7B

On cranial nerve examination, the patient was found to have bilateral partial cranial nerve III and IV palsies, with an inability to gaze downward in either adduction or abduction, as well as moderate weakness of the bilateral orbicularis oculi muscles and mild tongue weakness. Muscle tone in all four extremities was equally flaccid. Muscle strength was decreased proximally more than distally in all extremities, affecting the upper and lower limbs similarly. Reflexes were absent in all tested extremities, including biceps, brachioradialis, triceps, and ankles, except for a slight (1+) flicker in the bilateral patellar reflexes. All other physical exam maneuvers, including light touch, temperature, vibration sensation, and ataxia testing, were without gross abnormalities.

The combination of progressive weakness, cranial nerve III and IV palsies, reduced reflexes, and behavioral changes prompted a broad differential diagnosis. Subsequent workup included a serum paraneoplastic antibody panel, lactate dehydrogenase, creatine kinase, aldolase, voltage-gated calcium channel antibody, a 211-gene neuromuscular panel (Table 1, Table 2), MRI of the brain with and without contrast, and electromyography/nerve conduction study (NCS).

Sensory NCS of the right upper and lower extremities (ulnar digit five, superficial radial, sural, and superficial peroneal nerves) and motor NCS (ulnar, peroneal, and tibial nerves) were significant only for findings suggestive of carpal tunnel syndrome. Needle EMG of the right upper and lower extremities (tibialis anterior, gastrocnemius, vastus medialis, tensor fasciae latae, iliopsoas, first dorsal interosseous, flexor carpi radialis, triceps brachii, deltoid, and masseter) showed only myopathic motor units in the abductor pollicis, consistent with the aforementioned carpal tunnel findings.

Genetic testing revealed a homozygous pathogenic variant in ATP7B, with subsequent laboratory testing showing significantly decreased serum ceruloplasmin and elevated 24-hour urine copper (Table 1, Table 2). MRI of the brain, per the radiologist’s report, demonstrated dilated ventricles consistent with hydrocephalus ex vacuo from cerebral atrophy (images not available from the outside facility). The patient was started on penicillamine for presumed Wilson’s disease, titrated from 250 mg daily to a planned goal dose of 500 mg twice daily.

At follow-up one and a half months later, the patient demonstrated significant subjective and objective improvement in both ophthalmoplegias and muscle strength. Progress was limited by nonadherence: he took penicillamine 250 mg daily for one week, then 500 mg daily for two weeks, before running out of medication for one week. Resumption at 500 mg daily led to the development of fevers. The workup for infection was negative, and a drug-related reaction was diagnosed. Due to disruption of the titration schedule and subsequent fevers, the patient was started on zinc acetate while pending insurance approval for trientine. The patient was eventually lost to follow-up over the subsequent three months, and further documentation was unavailable.

## Discussion

Wilson’s disease exhibits an exceptionally broad spectrum of clinical presentations, with only a small subset of patients demonstrating primary skeletal muscle involvement. In our case, the diagnosis was delayed by several months because initial evaluations focused on alternative neuromuscular etiologies and on comorbid conditions, such as cirrhosis and severe anxiety, as isolated problems rather than as components of a unified systemic illness. The patient’s facial weakness, bilateral upper and lower extremity weakness, and diffusely diminished to absent reflexes pointed toward a myopathic process. Intact nerve conduction studies, aside from a median neuropathy from carpal tunnel syndrome, ruled out a primary nerve problem. A normal EMG did not exclude muscle pathology, since EMG predominantly assesses type I muscle fibers and is often less sensitive in disorders affecting muscle mechanics or in mitochondrial myopathies [5,6].

The combination of ophthalmoplegia and skeletal muscle weakness is well-described in inflammatory demyelinating polyneuropathies, neuromuscular junction disorders, and mitochondrial myopathies. In our patient, nerve conduction studies, clinical history, and antibody panels effectively excluded demyelinating disease and neuromuscular junction pathology. After an extensive workup, including large-scale genetic testing, MRI of the brain and full spine (with and without contrast), comprehensive serum studies (autoimmune, metabolic, nutritional, and infectious), CSF studies, and electrodiagnostic studies, the only significant findings were two pathogenic variants in ATP7B, decreased serum ceruloplasmin, and elevated 24-hour urine copper. The patient’s subsequent improvement on low-dose copper chelators confirms Wilson’s disease as the primary driver of his myopathy.

A proposed mechanism of myopathy secondary to Wilson’s disease involves oxidative stress-mediated skeletal muscle damage, in which free radical formation leads to mitochondrial deterioration and, consequently, cell death [1]. While the precise mechanism remains unclear, studies suggest that copper toxicity interferes with iron-sulfur cluster assembly in the mitochondrial matrix, impairing cellular respiration and ultimately triggering myocyte death [6,7].

A review of PubMed cases (search terms: “Wilson’s disease” AND “myopathy”, 2010-2025) yielded 32 articles, with only three reporting primary myopathy attributed to Wilson’s disease (Table 3). Although limited, each of these cases demonstrated some degree of improvement following treatment, supporting Wilson’s disease as the causative factor. Similarly, Taly et al.’s multicenter series of 282 patients with Wilson’s disease over three decades (1970-2000) identified only 22 patients (7.8%) with proximal weakness resembling myopathy [8]. Collectively, these data underscore the rarity of true Wilson-related muscle disease and highlight the importance of maintaining a high index of suspicion when systemic and neuromuscular features coexist.

**Table 3:** Wilson’s disease myopathy cases Cases of Wilson’s disease myopathy available on PubMed from 2010 to 2025 [1,9,10]. EMG, electromyography; MSK, musculoskeletal; NCS, nerve conduction study; TID, three times daily

Case	Age of symptom onset	Sex	MSK-related exam abnormalities	NCS/EMG	Therapy	Improvement of weakness with therapy?	Improvement noted at follow-up	Reference
1	8	Male	Bilateral hip extensor and abductor weakness (3/5)	NCS: normal; EMG: myopathic units of quadriceps femoris	Oral penicillamine 20 mg/kg/day; oral zinc 25 mg TID	Yes	Three months	[1]
2	10	Male	Lower extremity cramps	Not performed	Trientine (unspecified dosage)	Yes	Two months	[9]
3	21	Male	Upper and lower limb weakness (2/5; specific distribution unspecified)	Not performed	D-penicillamine 500 mg/day, titrated up to 500 mg BID; oral pyridoxine 20 mg/day; oral zinc 50 mg TID	Yes	Initial improvement noted (specific timing not specified); no recurrence at one-year follow-up	[10]

An alternative mechanism for myopathy in Wilson’s disease involves hypokalemic periodic paralysis secondary to proximal renal tubular acidosis (RTA), resulting from copper-induced renal damage [10]. In Case 3 (Table 3), the patient exhibited a non-anion gap metabolic acidosis, with a urine anion gap and alkaline urine pH consistent with type 1 RTA. Further evaluation using fractional excretion of potassium suggested involvement of both distal and proximal tubules [10]. Notably, prior to the diagnosis of Wilson’s disease, the patient had experienced episodic muscle weakness that improved with potassium supplementation. In distal RTA, impaired hydrogen ion excretion in the distal nephron leads to potassium wasting and systemic hypokalemia, a well-recognized cause of hypokalemic periodic paralysis. This mechanism is documented in the literature and offers a compelling secondary explanation for muscle weakness in select Wilson’s disease cases.

Although reported cases of Wilson’s disease-associated myopathy remain rare, the extensive exclusion of alternative diagnoses, both in the literature and in our case, combined with clinical improvement following copper chelation therapy, strongly supports a direct causative link between Wilson’s disease and myopathy. As additional cases are documented and mechanistic insights evolve, a clearer understanding of this rare manifestation is likely to emerge.

## Conclusions

Wilson’s disease should be considered a rare but important cause of idiopathic myopathy, particularly in the presence of comorbid conditions such as psychiatric symptoms and hepatic dysfunction, as seen in our case. Genetic testing should be regarded as an integral component of the workup for idiopathic myopathy when initial neurologic evaluations are unrevealing. Early recognition is crucial to prevent not only further neurologic damage but also systemic deterioration. Clinicians should remain vigilant to the possibility that seemingly unrelated comorbidities may, in fact, reflect a single underlying systemic disorder. Larger multicenter studies could be valuable in better characterizing Wilson’s disease-related myopathies.
