# Correction: Wilson’s Disease Myopathy: A Case Report and Brief Literature Review

**DOI:** 10.7759/cureus.c444

**Published:** 2026-06-02

**Authors:** Nicholas M Riccione, Cassie N Chan

**Affiliations:** 1 Neurology, University of Texas Health Science Center at San Antonio, San Antonio, USA

This article has been corrected to replace the following typo: the medication "trientine" was erroneously referred to as "triamterene" in the last paragraph of the Case Presentation section.

